# Albumin-Based Nanoparticles with Factorial Design as a Promising Approach for Remodeled Repaglinide: Evidence from In Silico, In Vitro, and In Vivo Evaluations

**DOI:** 10.3390/pharmaceutics17030350

**Published:** 2025-03-09

**Authors:** Mennatullah M. Faisal, Eman Gomaa, Mohamed S. Attia, Rana M. Abdelnaby, Adel Ehab Ibrahim, Ahmed Al-Harrasi, Sami El Deeb, Al Zahraa G. Al Ashmawy

**Affiliations:** 1 Department of Pharmaceutics, Faculty of Pharmacy, Zagazig University, Zagazig 44519, Egypt; mennafaisal@yahoo.com (M.M.F.); eman_pharmaceutics@yahoo.com (E.G.); mosalahnabet@gmail.com (M.S.A.); 2 Nanotechnology Research Center (NTRC), The British University in Egypt (BUE), Cairo 11837, Egypt; 3 Department Pharmaceutical Chemistry, Faculty of Pharmacy, Heliopolis University, Cairo 11785, Egypt; 4 Natural and Medical Sciences Research Center, University of Nizwa, Birkat Al Mauz, P.O. Box 33, Nizwa 616, Oman; aharrasi@unizwa.edu.om; 5 Institute of Medicinal and Pharmaceutical Chemistry, Technische Universität Braunschweig, 38106 Brunswick, Germany; 6 Department of Pharmaceutics, Faculty of Pharmacy, El Saleheya El Gadida University, El Saleheya El Gadida 44813, Egypt; zahraa_ashmawy@yahoo.com

**Keywords:** factorial design, atherosclerosis, albumin, nanoparticles, in silico, triglycerides, total cholesterol

## Abstract

**Background/Objectives:** Hyperlipidemia is a silent threat lurking in the bloodstream of millions worldwide. The nano-based platform has emerged as a promising drug delivery technology. Repaglinide, an anti-diabetic drug, was investigated recently as an antihyperlipidemic candidate that could supersede the available antihyperlipidemic drugs. Our goal was to optimize albumin-based nanoparticles loaded with Repaglinide for parenteral delivery and conduct in silico and in vivo studies to explore the efficacy of Repaglinide for the management of hyperlipidemia along with its anti-diabetic effect. **Methods:** The impact of three independent factors, the albumin%, acetone volume, and glutaraldehyde/albumin, on the particle size, zeta potential, and entrapment efficiency was investigated. **Results:** The optimized formulation was spherical, homogenous of an average diameter (~181.86 nm) with a narrow size distribution, a zeta potential of −24.26 mV, and 76.37% as the EE%. The in vitro release of Repaglinide from nanoparticles showed a sustained release pattern for 168 h, with an initial burst release after 24 h, and was fitted to the Fickian diffusion mechanism. A molecular docking simulation showed a strong affinity to several protein targets, and the results were very promising, where Repaglinide gave a score of −7.70 Kcal/mol compared to Mevastatin (−6.71 Kcal/mol) and Atorvastatin (−8.36 Kcal/mol). On conducting in vivo studies on animal models, the optimized formula recorded a statistically significant decrease in the serum levels of total cholesterol, triglyceride, and low-density lipoproteins, with an increased high-density lipoprotein. **Conclusions:** This study suggested albumin nanoparticles as potential nanocarriers for the parenteral delivery of Repaglinide to ameliorate its antihyperlipidemic benefits, especially in diabetic patients.

## 1. Introduction

Atherosclerosis, the deposition of fats and fibrous materials on the walls of blood vessels [1], is a well-known cardiovascular risk, may be one of type 2 diabetes mellitus’ serious consequences, and a leading cause of mortality all over the world [2]. According to the data obtained from the Center for Disease Control and Prevention (CDC), in the US, about 37.1% of people, which constitutes 73.5 million adults, are suffering from high blood levels of LDLs and are subsequently at high risk of cardiovascular diseases [3]. In addition, the economic burden of atherosclerotic cardiovascular diseases ranges from USD 17 to 259 million, according to studies conducted in the US, as mentioned by Ferrara et al., 2021 [4]. Statins have been widely used for the treatment of hypercholesterolemia to decrease the serum levels of total cholesterol (TC), triglycerides (TGs), and low-density lipoproteins (LDLs) while increasing high-density lipoproteins (HDLs) [5]. Despite being very effective medications for the treatment of hypercholesterolemia, statins possess very low oral bioavailability due to the extensive first-pass metabolism [6].

Moreover, and according to the American College of Cardiology, about 10% of hyperlipidemic patients in the USA stopped using statins as they suffer from muscle pain, weakness, cramps, and spasms as a result of myopathy caused by statins [7]. As a result, the FDA added warning labels to statin medications on the market based on their side effects, such as dementia, memory loss, and mental confusion [8]. Many alternatives for statins, such as fibrates, plant stanols, red yeast rice extract, omega 3, bile acid-binding resins, policosanol, niacin, and others, have emerged for the management of hyperlipidemia. Unfortunately, none of them compete with statins, which are still considered the best class of drugs used for the treatment of hyperlipidemia [9,10].

Repaglinide is a class II anti-diabetic drug, approved by the FDA in 1998 for the treatment of diabetes mellitus type 2 as it stimulates the release of insulin [11]. Recently, it was introduced to elicit a pivotal role in the management of hypercholesterolemia by reducing TC, TG, and LDLs [12]. When administered orally and as a result of the extensive first-pass metabolism, Repaglinide possesses a very poor oral bioavailability of less than 55%, with a short half-life of about 60 min [13]. As a result, it is administered orally two to three times per day before every meal, which negatively satisfies the patient [11]. So, developing an injectable nano-delivery system of a high stability, high loading capacity, and with the feasibility of incorporating both hydrophilic and hydrophobic candidates for variable administration routes would be effective to ameliorate its clinical effect with more patient compliance [14].

Nanoparticles have been shown to provide many advantages over conventional drug delivery systems and have proven to be an effective approach for drug targeting [15]. Specifically, albumin is considered a unique carrier of nanoparticles, as it has a strong ability to solubilize both hydrophilic and hydrophobic drugs, offering a long half-life, and achieve drug targeting with lower toxicity [16]. Bovine serum albumin (BSA) and human serum albumin (HSA) are two distinctive sorts of albumin, possessing high water solubility with a long half-life; thus, they were introduced as privileges in nanoparticle preparation [17]. BSA has been widely used as a drug carrier, especially in injectable dosage forms, owing to its biocompatibility, safety, and its non-antigenic properties. Encapsulating Repaglinide in simply prepared and cost-effective albumin-based nanoparticles seems to be a good approach to improve its limited bioavailability, overcome rapid elimination, sustain its release, decrease the frequency of administration, and, as a result, improve patient compliance. The current study aimed to optimize albumin-based nanoparticles loaded with Repaglinide, using 2^3^ full-factorial design. The optimized formulation was characterized for particle size, differential scanning calorimetry (DSC), scanning electron microscopes (SEMs), transmission electron microscopes (TEMs), and in vitro drug release. The antihyperlipidemic effect of Repaglinide was investigated by an in silico study for the determination of the interaction of Repaglinide with target proteins and confirmed through a rat model. Measurement of the serum cholesterol and triglyceride levels was performed to confirm its efficacy as an alternative to statins for the proper management of hyperlipidemia in addition to its antihyperglycemic effect. This approach could be effective, especially in diabetic patients developing hyperlipidemia, without the hazards of using statins.

## 2. Materials and Methods

### 2.1. Materials

Repaglinide was kindly gifted by the Egyptian International Pharmaceutical Industries Co. (EIPICO, 10th of Ramadan, Egypt). Glutaraldehyde was purchased from Loba Chemie (Mumbai, Maharashtra, India), and bovine serum albumin was purchased from Sigma-Aldrich (St. Louis, MO, USA). Disodium hydrogen orthophosphate, potassium dihydrogen orthophosphate, triethyl amine, sucrose, and acetone were supplied by El-Nasr Pharmaceutical Chemicals (Abu-Zaabal, Cairo, Egypt). All chemicals were of high analytical grades.

### 2.2. Experimental Design

A 2^3^ full factorial design was constructed by Design-Expert^®^ software (version 13, Stat-Ease Inc., Minneapolis, MN, USA) to study the relationship between the independent factors and dependent responses. Albumin % (A), acetone volume (B), and 1 µL glutaraldehyde per 2 mg albumin (C) were set at two different levels, (+1) and (−1), as the three independent factors studied. Three dependent responses were evaluated, namely particle size (nm) (Y1), zeta potential (−mV) (Y2), and entrapment efficiency % (Y3). The two levels of the independent factors, and the goals required for the dependent responses, are represented in Table 1. In addition, Table 2 displays the eight experimental runs that were suggested by the design software under study.

### 2.3. Preparation of Repaglinide-Loaded Albumin-Based Nanoparticles

Repaglinide-loaded albumin-based nanoparticles were prepared by the desolvation technique according to the method depicted by Chen et al. (2020) and Storp et al. (2012) [18,19], with certain modifications. In brief, BSA at two different concentrations (0.5 and 1%) was dissolved in exactly 5 mL of distilled water, and the pH of the prepared solution was adjusted to 7.4 using triethyl amine. Exactly, an amount of 4 mg of Repaglinide was dissolved in BSA solution, and then acetone (desolvating agent) was added dropwise to the polymeric solution, containing Repaglinide, under continuous stirring using a magnetic stirrer (Type MM5, Poznan, Poland) at 1500 rpm. The stirring forces the aggregation and enhances the formation of nanoparticles [12,20]. The nanoparticles were finally completely formed via the addition of glutaraldehyde, the crosslinking agent, at a volume of 1 or 3 µL to each 2 mg of BSA within about 2 h. The acetone content was then evaporated from the mixture using the Basis Hei-VAP rotary evaporator from Heidolph Instruments GmbH (Schwabach, Bavaria, Germany) under a negative pressure of −10 pa at 35°C and 100 rpm. Finally, the prepared nanoparticles were dispersed in distilled water, and the dispersion was sonicated by a probe sonicator (Model GE 50, from Scientific Engineering Inc., Woodbridge, VA, USA) for 2 min at 50 kHz/room temperature. The prepared formulations were then lyophilized by the use of a freeze dryer (Heto Power Dry LL1500-Thermo Electron Corporation, Waltham, MA, USA) and stored at 4°C for further investigations [18]. To prevent the irreversible aggregation of the nanoparticles, 10% sucrose was added as a lyoprotectant to all of the prepared formulations [21,22].

### 2.4. Evaluation of Repaglinide-Loaded Albumin-Based Nanoparticles

#### 2.4.1. Particle Size, Polydispersibility Index, and Zeta Potential

The particle size (PS), polydispersibility index (PDI), and zeta potential (ZP) of Repaglinide-loaded albumin-based nanoparticles were measured by dynamic light scattering and laser Doppler anemometry techniques using a Malvern Zeta sizer (Nano ZS, Malvern Co., Malvern, UK) using a clear disposable zeta cell, as described by Wu et al., 2020 [23]. All formulations were diluted using double-distilled water before being measured at 25°C. Values were presented as a means of triplicate measurements ± standard deviation (SD).

The ZP of any particle describes the net charge on the particle in a certain medium, and this could help assess stability amid the storage stage. PDI is a ratio used for the estimation of the homogeneity of particle size distribution in a colloidal system. A PDI value less than 1 indicates a homogenous preparation with an acceptable size distribution [24].

#### 2.4.2. Entrapment Efficiency %

The entrapment efficiency of a nanoparticle (EE%) is a measure of the ability of the vesicles to encapsulate the drug in relation to the total amount of drug used [25]. The prepared formulations were separately packed in previously soaked dialysis bags (Mwt of 12,000–14,000 Da). Then, the dialysis bags were immersed in a 250 mL beaker containing 100 mL of Sörenson’s phosphate buffer (SPB, pH 7.4). The entire assembly was kept in a thermostatic Kotterman shaker water bath (from Kotterman GmbH, Uetze, Germany) adjusted at 100 rpm and 25 °C ± 0.5 °C. After one hour, the amount of free drug was separated using a 0.22 µm nylon syringe filter and measured spectrophotometrically at λ max of 242 nm [17,26]. The entrapment efficiencies of all formulations from F1 to F8 were calculated using the following equation:

$$Entrapment\;efficiency\;percentage=\frac{Total\;initial\;drug\;amount-Free\;drug\;amount}{Total\;initial\;drug\;amount}\times 100$$

#### 2.4.3. In Vitro Drug Release

The in vitro release of Repaglinide from the prepared formulation was performed through a dialysis bag (Spectrum Labs^®^ MWCO, 7000 Da; from Fischer Scientific, Waltham, MA, USA). The dialysis bags were immersed into glass bottles containing 50 mL of SPB solution (pH 7.4), which were kept in a thermostatic shaker water bath (Kotterman shaker, from Kotterman GmbH, Uetze, Germany), adjusted at 37 ± 0.2 °C and 100 rpm. At time intervals of 1, 2, 4, 8, 24, 48, 72, 96, 120, 140, and 160 h, a 3 mL sample of the SPB solution was withdrawn, filtered through a 0.22 µm nylon syringe filter, and then assayed spectrophotometrically at λ_max_ 242 nm for its Repaglinide drug content. After each sampling, an equal volume of fresh buffer was added to maintain the sink conditions. All of the assay measurements were represented as a mean of triplicate experiments ± standard deviation [27].

#### 2.4.4. Drug Release Kinetics

The results of the drug release were analyzed based on different kinetic models, namely zero-order, first-order, Higuchi, Hixson–Crowell, and Korsmeyer–Peppas models. This was carried out to determine the model that best fits the release pattern of the drug, based on the values of the correlation coefficient (R^2^). The model with the highest R^2^ was selected as the most accurate model describing Repaglinide’s release from albumin-based nanoparticles. The Korsmeyer–Peppas model’s *n* values were used to confirm the drug release mechanism from the formulations under study.

### 2.5. Optimization and Statistical Validation

Based on the selected criteria of maximizing EE% and ZP values, while minimizing PS, an optimized formulation was proposed by Design-Expert^®^ software (version 13, Stat-Ease Inc., Minneapolis, MN, USA). The suggested formulation was prepared and characterized again to compare the correlation between the experimental Y1–Y3 response values and those predicted by the design.

### 2.6. Transmission Electron Microscopy (TEM)

The optimized formula of Repaglinide was analyzed for its morphological characters using TEM (JEOL GEM-1010, Tokyo, Japan). One drop of the preparation was dispersed in distilled water and added onto a carbon coated grid. After being kept for 2 min, a filter paper was applied to dry any excess liquid. A drop of a 2% aqueous solution of phosphotungstic acid was added to the sample, and then it was left to dry at room temperature. The sample was then ready for examination under TEM at an accelerating voltage of 80 kV [28].

### 2.7. Scanning Electron Microscopy (SEM)

SEM was used to illustrate the morphology, surface characters, and shape of the prepared Repaglinide nanoparticles. Gold was used for coating the prepared nanoparticles using a voltage of 7 kV [29].

### 2.8. Differential Scanning Calorimetry (DSC)

The DSC analyzer (DT-60 DSC, from Shimadzu, Kyoto, Japan) was used to detect the thermal behaviors of pure Repaglinide, BSA, their physical mixture, as well as the thermal behavior of the optimized formulation. Samples were placed into sealed aluminum pans, where they were subjected to direct temperatures ranging from 0 to 200 °C at a rate of 10 °C/min. The samples were kept under nitrogen atmosphere at a rate of flow of 20 mL/min [30].

### 2.9. Molecular Docking

Molecular modeling was performed to study the binding interactions between the drug and the type of protein under the current investigation. Firstly, the 3D crystal structures of the nominated proteins were obtained from the data bank for proteins (HMG-CoA; protein data bank (PDB) code: 1HW8, ACAT; PDB: 7N6R, and NPC1L1; PDB: 6V3H). The 3D crystal structures were prepared by removing the water molecules, repairing the shortened side chains, adding hydrogen, imposing partial charges, and applying the energy minimization protocol. The structure of Repaglinide was obtained from the Pubchem database (United States National Institutes of Health, https://pubchem.ncbi.nlm.nih.gov/; accessed on 28 February 2025), and the energy was minimized for docking by adding hydrogen and assigning charges and other force field parameters. Molecular docking was performed, and the results were measured as binding affinities in kcal/mol. The 2D and 3D binding modes of the best poses were visualized and studied using the Discovery Studio visualizer (San Diego, CA, USA).

### 2.10. In Vivo Evaluation of the Optimized Formulation

Repaglinide’s antihyperlipidemic effect was examined according to a reference antihyperlipidemic drug, atorvastatin calcium (ATC), to determine Repaglinide’s effectiveness for the treatment of hyperlipidemia along with its anti-diabetic effect.

Exactly 30 adult male albino rats weighting 200 ± 50 g were included in the experiment [31]. Before the initiation of the study, the rats were maintained in a 12 h light–dark cycle at room temperature for a week, with free access to water and food. The proposed study followed the Faculty of Pharmacy’s Institutional Animal Care and Use Committee (IACUC) guidelines (Approval No.: ZU-IACUC/3/F/5/2024).

Rats were equally distributed into five groups (*n* = 6); the first group served as a negative control (without induction of hyperlipidemia). The induction of hyperlipidemia in the other four rat groups was achieved by the oral ingestion of a high-fat diet consisting of 60% of their normal food mixed with 20% margarine and 20% sugar, as well as the oral feeding of rats with 2 mL of sesame oil mixed with cholesterol for one week [32]. The five groups were classified as follows:

Group I represented a normal group without the induction of hyperlipidemia. Group II represented hyperlipidemic rats who received no treatment (a positive control group). Group III represented hyperlipidemic rats who received an oral suspension of pure ATC at a dose of 3 mg per kg of animal weight [33]. Group IV received an oral suspension of pure Repaglinide in a dose equivalent to 1 mg/kg [12]. Group V received a single intramuscular injection (IM) of optimized Repaglinide nanoparticle formulation (F5) suspended in a phosphate buffer (pH 7.4) in a dose equivalent to 1 mg/kg per day [12].

All of the treated rat groups received the treatments for one week using an oral gavage (Instech Laboratories Inc., Plymouth Meeting, PA, USA), except for Group V, as the rats in this group were injected intramuscularly by the treatment [32].

Blood cholesterol levels, TC, TGs, LDLs, and HDLs, were measured using diagnostic kits (Spin React, Barcelona, Spain). Blood samples were collected from the rat’s lateral tail veins after their treatment according to the protocol mentioned before, where 1 ml of blood was collected from each rat.

A one-way ANOVA test was applied to statistically interpret the results using GraphPad Prism 8.0.1, San Diego, CA, USA. The level of significance was set as a *p*-value of <0.05.

## 3. Results and Discussion

### 3.1. Repaglinide-Loaded Albumin-Based Nanoparticles

In the current study, Repaglinide was developed as an injectable nanoparticle by using a safe, biocompatible, and biodegradable polymer: BSA. Throughout the desolvation process, the nanoparticles were generated by adding acetone to the BSA aqueous solution at a high pH until a turbid solution was formed. Acetone, as a solvent, has low toxicity (class 3) and possesses a low risk level regarding human health. The stability of BSA nanoparticles was low and may be subjected to phase reversal. That is why glutaraldehyde was added during the formulation of BSA nanoparticles, as a crosslinking agent, to promote their stability by a condensation reaction between the side chained amino groups of BSA and the aldehyde groups of glutaraldehyde [34]. It is noteworthy that the preparation method of BSA nanoparticles should be carried out at a high pH, above the isoelectric point of albumin (4.9), in order to increase the magnitude of BSA negative charges and prevent the agglomeration of nanoparticles, and hence improve stability [17].

### 3.2. Experimental Design and Statistical Analysis

Eight nanoparticle formulations were suggested by 2^3^ full factorial design, based on the selected three independent variables (A, B, and C) at two different levels of high and low values. After preparation, the formulations were assessed and the corresponding dependent responses, particle size (Y1), zeta potential (Y2), and entrapment efficiency (Y3), were measured and statistically analyzed.

#### 3.2.1. Effect of Independent Variables on the Particle Size of Nanoparticles (Y1)

The particle size of the Repaglinide-loaded albumin nanoparticles formulae (F1–F8) was measured and found to be in the range between 136.63 ± 0.55 and 217.83 ± 0.43 nm (Table 2). The statistical analysis of the model revealed that the particle size of different formulations was significant, with an F value of 4885.66 and a *p*-value of < 0.0001, as shown in Table S1. Additionally, factors A, B, and C were all significant in terms of their *p*-values (<0.0001). The R^2^ was of a high value (0.9994), and the precision value was 204.154, indicating an adequate signal/noise ratio, and the model can successfully navigate the design space.

**Figure 1:**
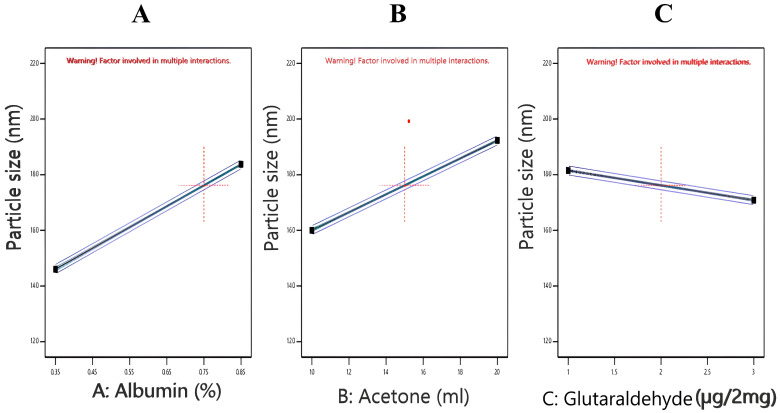
One factor plots of Y1 response (particle size). (**A**) Effect of A (albumin %) on Y1; (**B**) effect of B (Volume of acetone) on Y1; (**C**) effect of C (µL glutaraldehyde per 2 mg of albumin) on Y1.

The effect of the interaction between variables on particle size is illustrated in Figure 1. The polynomial equation by the Y1 model is as follows:

Y1 = 176.171 + 18.8292 × A + 16.1375 × B − 5.34583 × C − 1.2375 × AB + 0.495833 × AC − 2.79583 × BC.

It was clear that there was an interaction between the two factors, B and C, while there was no interaction between factors AB and AC that could affect particle size (parallel lines). It was obvious that the lower the concentrations of BSA, the smaller the particle size, and vice versa (Figure 1A). This is illustrated on the basis that increasing the BSA concentration is accompanied by an increase in viscosity. The increase in viscosity negatively affected the rate of protein transfer between water and the desolvating agent. The rate of nucleation was subsequently decreased, producing larger particles as a result of nanoparticles’ coagulation. These results were in agreement with those obtained by Attia et al., 2023 [17]. Inan and Özçimen, 2021, and Esim et al., 2021, suggested that certain types of electrostatic and hydrophobic interactions could occur between nanoparticles as a result of their coagulation, which was elicited when a higher percentage of BSA was used [35,36]. Regarding the effect of acetone, the increase in the acetone concentration was accompanied by a significant increase in particle size (Figure 1B). This could be attributed to the fact that the increase in the acetone concentration could lead to the aggregation of the spherical particles where larger clusters are formed [37]. Regarding the crosslinking agent (glutaraldehyde), the increase in its amount led to a significant decrease in particle size (Figure 1C), and these results were in accordance with those found by Radwan et al., 2022 [38], and Nguyen et al., 2020 [39], who declared that increasing glutaraldehyde from 1.25 to 5 decreased the particle size significantly from 120 to 80 nm. It is noteworthy that PDI of all the prepared formulations ranged between 0.112 and 0.277, indicating their high stability and homogeneity, which could help their proper absorption inside the body after being injected in vivo [15].

#### 3.2.2. Effect of Independent Factors on the Zeta Potential of Nanoparticles (Y2)

The zeta potential is defined as the charge intensity of the nanoparticles, and it is a very crucial parameter indicating the electrostatic interaction between nanoparticles. This, in turn, helps to assess the formulations’ stability during their storage and subsequently their shelf life on the market [17]. A higher zeta potential, above 20 mV, is recommended because it gives rise to lesser aggregates and subsequently a more stable preparation [40]. The results of the ANOVA statistical analysis of the model revealed that the zeta potential of the different formulations was significant (Table S1), with an F value of 12.44 and a *p*-value of <0.0001. In addition, A, C, and BC were considered significant terms with *p*-values <0.05. There was only a significant impact of BC on ZP. In this model, the R^2^ value was found to be as high as 0.8145, while the adequate precision value was above 4 (9.841), which gave rise to the model’s capability to navigate the design space. The polynomial equation for the Y2 model is as follows:

Y2 = −26.7708 + 1.5625 × A − 0.0625 × B − 1.57917 × C − 0.3125 × AB − 0.279167 × AC − 0.770833 × BC

**Figure 2:**
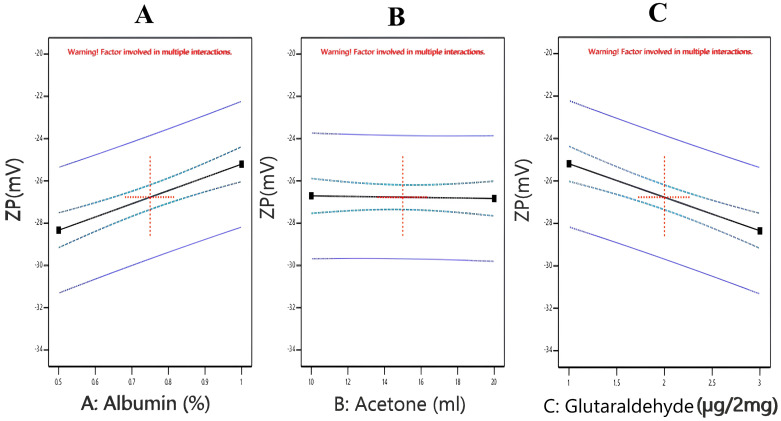
One factor plots of Y2 response (zeta potential). (**A**) Effect of A (albumin %) on Y2; (**B**) effect of B (Volume of acetone) on Y2; (**C**) effect of C (µL glutaraldehyde per 2 mg of albumin) on Y2.

As shown in Table 2, the zeta potential of the prepared nanoparticles ranged between −22.43 and −29.63 mV. These results indicated the stability of all the prepared nanoparticles with a low tendency for aggregation and subsequently more stable formulations. The negative values of the zeta potential might be attributed to the presence of BSA at a pH higher than the isoelectric point. As we previously mentioned, the pH was maintained between 8 and 9, and at such pH values, the value of the zeta potential became negative [41]. Moreover, due to its high solvent polarity, acetone also contributed to such a negative charge of the zeta potential [42]. Meanwhile, the results obtained revealed a significant negative correlation of ZP with the glutaraldehyde concentration. Similar outcomes were obtained by Khan et al., 2011 [43], who illustrated that the zeta potential of the nanoparticles decreased as the concentration of glutaraldehyde increased. On the contrary, acetone showed an insignificant effect on the zeta potential. Meanwhile, there was a significant positive correlation of the zeta potential with BSA, where the zeta potential increased by increasing BSA (Figure 2). These results were in good alignment with the results obtained by Mikani et al., 2018 [44], who found that ZP changes might be related to the negative surface charge of the BSA molecules, which resulted in a spontaneous increase in the electrostatic repulsion between the nanoparticles.

#### 3.2.3. Effect of Independent Factors on Entrapment Efficiency of Nanoparticles (Y3)

**Figure 3:**
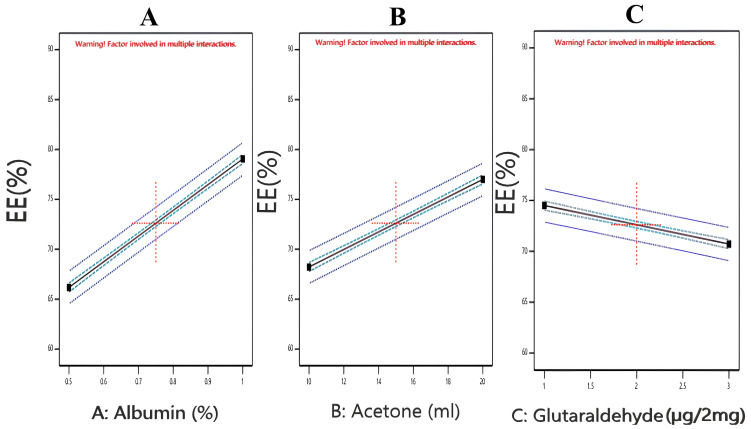
One factor plots of Y3 response (entrapment efficiency%). (**A**) Effect of A (albumin %) on Y3; (**B**) effect of B (Volume of acetone) on Y3; (**C**) effect of C (µL glutaraldehyde per 2 mg of albumin) on Y3.

The values of the entrapment efficiency of the prepared nanoparticles varied from 61.03% ± 0.64 to 86.90% ± 1.04 (Table 2). The impact of the three factors on EE% was studied individually (Figure 3). The statistical analysis of the model revealed that the entrapment efficiencies of the different formulations were significant, with an F value of 479.11 and a *p*-value of < 0.0001. The three studied factors (A, B, and C) were significant in terms of their *p*-values (<0.0001). Moreover, the terms AB, AC, and BC showed a significant impact on EE% (Y3 response). The model showed a high R^2^ (0.9941), as well as a high precision value (63.1831), which indicated an adequate signal. The polynomial equation developed by the Y3 model is as follows:

Y3 = 72.6125 + 6.4375 × A + 4.3875 × B − 1.89583 × C + 1.22917 × AB − 0.6875 × AC + 0.5625 × BC

Figure 3 demonstrates the effect of each factor on EE%. EE% increased with increasing BSA and acetone concentrations. Increasing EE% with an increasing albumin concentration could be attributed to increasing the availability of albumin molecules around the drug, which can help increase the compactness of the particles with better EE% [45]. These results were in agreement with those obtained by Attia et al., 2023, who observed that upon increasing the concentration of BSA, the entrapment efficiencies of all their formulations increased [17]. The effect of acetone on EE% could be explained by the fact that any increase in the acetone concentration can result in larger nanoparticles, which enhanced the encapsulation of more drug molecules. On the other hand, glutaraldehyde showed a decrease in EE% when being incorporated at increasing concentrations, and this result was in alignment with that obtained by Sethi et al., 2021 [46].

#### 3.2.4. In Vitro Drug Release and Release Mechanism

**Figure 4:**
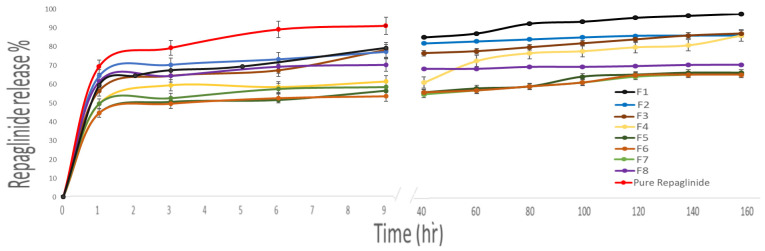
In vitro drug release of Repaglinide from BSA nanoparticles (F1–F8) in SPB solution (pH 7.4) at 37 ± 0.2 °C and 100 rpm for 160 h.

The in vitro release of the prepared Repaglinide nanoparticles after 168 h is illustrated in Figure 4. The release profile of the pure drug showed an initial burst effect during the first 2 h, which was followed by an increase in the release rate to reach 90% after 24 h. The same results were mentioned by Nanjwade et al., 2013 [47]. The release profile of all of the formulations was found to have two phases: an initial phase, which constitutes burst drug release for the first 24 h, and then sustained release of the drug, which was completed until 168 h. These results indicated the availability of Repaglinide to produce sustained release effects for the long-term treatment of hyperlipidemic patients with no need for frequent drug administration, which in turn improves patient compliance. On increasing the BSA concentration, the release of the drug became much slower, and a more sustained release effect was achieved. BSA is responsible for the controlled release pattern of Repaglinide by hindering fast drug release. In addition, the action of glutaraldehyde, which was chosen as a crosslinking agent, contributed to the slow release of Repaglinide [48]. The release profile was observed in two phases. The initial phase represents a burst release of the drug at the first 24 h as a result of the release of the drug found close to the surface of the nanoparticles. Then, it was followed by a second phase of the sustained release of the entrapped drug until 168 h (7 days) [49]. The slowest drug release after 160 h was observed from F5, F6, F7, and F8, as they were composed of high concentrations of BSA in comparison to F1, F2, F3, and F4 (Figure 4). On the contrary, smaller nanoparticles possess a larger surface area, and thus the drug is present on their surfaces, leading to faster drug release, and this was obvious in F1, which showed a faster drug release of 99% after 168 h [49].

The in vitro release data were analyzed by applying mathematical kinetic models to elucidate the underlying mechanism of Repaglinide’s release from the nanoparticles, and the kinetic model with the highest R^2^ was chosen to accurately describe the drug release mechanism. The results in Table S2 depict that higher R^2^ values were relative to the Korsmeyer–Peppas model than other models. Based on the equation’s coefficient “*n*” of the Korsmeyer–Peppas model, the matrix’s type of release could be indicated. If *n* ≤ 0.43, this means a drug release through a diffusion mechanism, but if *n* ≥ 0.85, this indicates a drug release primarily through a matrix erosion process. Furthermore, if *n* values were between the mentioned ranges, this determines an anomalous mechanism, and a combination of both diffusion and erosion mechanisms as well. The results showed that the *n* values of the formulations were less than ≤ 0.43, which suggests that Repaglinide’s release from the prepared BSA-based nanoparticles fitted the Fickian diffusion mechanism [50,51]. On the contrary, the release of Repaglinide from pure suspension followed the first-order kinetics, indicating the dependence of the drug release on drug concentration, where the greater the concentration, the faster the process [52,53].

#### 3.2.5. Optimization Process

The optimization was carried out to find a formula that could achieve the goal of this study. The necessary criteria were minimum particle size along with maximum ZP and EE%. To achieve the aim of our study for preparing the BSA-based nanoparticles with the targeted characteristics, according to Table 1, the design software suggested an optimized nanoparticle formulation with a desirability value of 0.600 (F5). The optimized formulation is composed of a 1% BSA concentration, 10% acetone, and a glutaraldehyde concentration of 1 µg/2 mg albumin. This optimized formula had minimized Y1 and maximized Y2, as well as Y3. The values of the three responses, as predicted by the software, were 182.15 nm for Y1, −23.74 mV for Y2, and 76.58% for Y3, whereas the experimental findings were 181.86 nm, −24.26 mV, and 76.37% for the three studied responses, respectively (Table 2). The feasible consistency between the experimental and the predicted values could reflect the validity of the models.

### 3.3. In Vitro Evaluation of Optimized Repaglinide-Loaded Nanoparticle Formulation

#### 3.3.1. TEM Imaging

**Figure 5:**
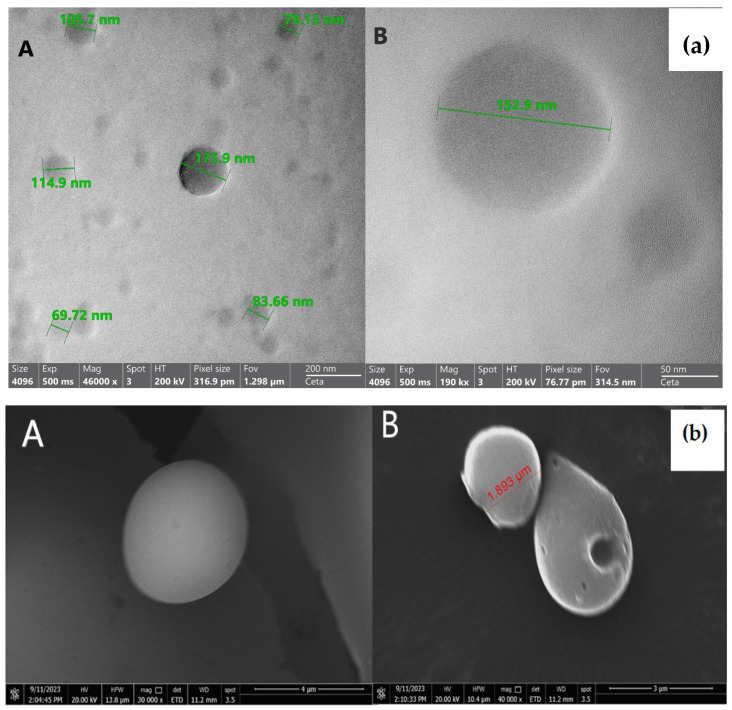
(**a**) TEM of the optimized Repaglinide nanoparticles: (F5) (**A**) 200 nm scale; (**B**) 50 nm scale. (**b**) SEM of the optimized Repaglinide nanoparticles: (F5) dried on aluminum foil (**A**) 4 µm scale; (**B**) 3 µm scale.

As shown in Figure 5a, the morphology of the optimized Repaglinide nanoparticles (F5) under TEM revealed that particles were homogenous and spherical in shape; similar results were obtained by Demirturk et al., 2025 [54]. The particle size of the nanoparticles, as illustrated by TEM, was found to be lower than their size range illustrated in the particle size measurement by Malvern Zetasizer. This could be attributed to the fact that the TEM imaging was carried out on the dried nanoparticles, while the measurement of the particle size on Zetasizer was performed in a wet state, which provides a slight fake increase in their particle size due to the water content [55].

#### 3.3.2. SEM Imaging

For a further investigation of Repaglinide nanoparticles, SEM imaging was performed in order to investigate their surface characters [56]. Figure 5b revealed that F5 nanoparticles were spherically shaped particles with smooth surfaces, showing the absence of drug crystals from their surface, which may confirm the complete entrapment of Repaglinide inside the nanoparticles.

#### 3.3.3. DSC Study

**Figure 6:**
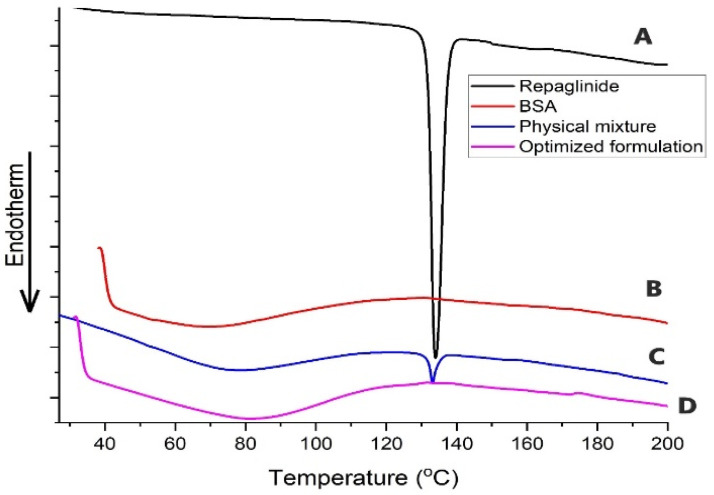
DSC of (**A**) pure Repaglinide, (**B**) BSA, (**C**) physical mixture of Repaglinide, and (**D**) optimized Repaglinide nanoparticles (F5).

The thermal behaviors of pure Repaglinide, BSA, their physical mixture, and the optimized formulation (F5) were tested using DSC. As shown in Figure 6, the DSC of pure Repaglinide demonstrates the presence of an obvious sharp endothermic peak at 134.02 °C, illustrating the drug’s crystallinity [57]. The DSC of BSA showed a broad endothermic peak at 79.53 °C, which appeared as a result of heating the protein more than its denaturation temperature [17]. The physical mixture of Repaglinide as well as BSA revealed a sharp endothermic peak with lower intensity and less melting enthalpy than the pure drug (132.96 °C), and this suggests that the crystalline Repaglinide was altered to an amorphous state when mixed with BSA [58]. Finally, the DSC of the optimized Repaglinide nanoparticles showed a complete absence of the endothermic peak of the pure drug due to the change in its crystalline nature into an amorphous state as a result of incorporating Repaglinide inside the protein structure of the nanoparticles [17].

### 3.4. Molecular Docking of Repaglinide

Repaglinide displayed a quite promising antihyperlipidemic effect. Molecular docking between Repaglinide and BSA was best described by Pawar and Jaldappagari, 2019 [59]. A literature survey revealed several well-reported proteins in the intestine and the liver that could be targeted for the treatment of hyperlipidemia. They are known as β-hydroxy β-methylglutaryl-CoA (HMG-CoA), Niemann-Pick C1-like-1 (NPC1L1) protein located at the apical membrane of intestinal enterocytes, peroxisome proliferation-activated receptors (PPARs), acyl-CoA cholesterol acyl transferase (ACAT), lipoprotein lipase, hepatic triglyceride lipase, and cholesterol ester transfer protein (CETP) [15]. In silico studies were carried out on HMG-CoA reductase, ACAT, and NPC1L1 as representative targets in the lipid metabolism.

#### 3.4.1. HMG-CoA Reductase Study

HMG-CoA reductase is a very important enzyme in the cholesterol synthesis pathway [60]. By comparing the docking results represented in Figure S1a, it was found that Repaglinide interacted in a similar way to both of the reference drugs, Mevastatin and Atorvastatin, which are known to be potent HMG-CoA inhibitors. Repaglinide formed hydrogen bonds with Asn A:658, Ser A:684, Lys A:692, and Lys B:735. Also, it formed van der Waals interactions with Asp A:690. Moreover, it was bound via the pi–alkyl interaction with Cyst B:561, Leu B:562, His B:752, and Leu B:853. The docking scores were very promising, where Repaglinide gave −7.70 Kcal/mol compared to Mevastatin with a score of −6.71 Kcal/mol and Atorvastatin with a score of −8.36 Kcal/mol.

#### 3.4.2. Acyl-CoA: Cholesterol Acyltransferases (ACAT2) STUDY

The endoplasmic reticulum (ER)-localized Acyl-CoA: cholesterol acyltransferase isoforms (ACAT1) and ACAT2 are among the membrane-bound O-acyltransferase family. They play a pivotal role in the homeostasis of cholesterol by activating the transfer of fatty acyl groups to the cholesterol’s 3b-hydroxyl group that are stored in either cytosolic lipid droplets or incorporated into LDLs and secreted from cells. Thus, molecules that can inhibit this enzyme will be beneficial in lowering hypercholesterolemia and the treatment of atherosclerosis and other cardiovascular diseases [15,61]. In this in silico study, Repaglinide was docked into the active site occupied by the co-crystalized inhibitor Nevanimibe. The resulting data was very promising, with a docking score of −8.74 Kcal/mol compared to Nevanimibe with a score of −7.48 Kcal/mol. Moreover, the binding interactions were as reported in the literature. Repaglinide formed a hydrogen bond with Asn395, while it formed pi–sigma interaction with Met449, and pi–alkyl interactions with Leu481 and Ile354, as presented in Figure S1b.

#### 3.4.3. Niemann-PickC1 Like 1 (NPC1L1) Study

NPC1L1 is a highly expressed enzyme on the apical membrane of intestinal enterocytes. It plays a pivotal role in dietary sterol uptake. Moreover, it is found on the canalicular membrane of hepatocytes that are responsible for the reabsorption and enterohepatic recycling of different sterols. Cholesterol molecules were reported to be transported to the plasma membrane via the continuous tunnel formed in the NPC1L1 closed form. The drug Ezetimibe was found to have a unique interaction with NPC without competing with cholesterol. It binds to the middle of this tunnel, occluding it without occupying the cholesterol binding pocket or overlapping with the cholesterol molecules found in the structure of NPC1L1apo-form, as shown in Figure S1c [15,62].

Since Repaglinide is an oral hypoglycemic drug, one of its suggested targets may be the NPC1L1 found in the intestinal wall. Thus, our team carried out docking studies on this enzyme. The docking scores were comparable to the reference inhibitor, Ezetimibe, giving −8.75 Kcal/mol, and interacting with the key amino acids Tyr472, Phe532, Met543, and Pro1021.

### 3.5. In Vivo Antihyperlipidemic Evaluation of Optimized Repaglinide-Loaded BSA Nanoparticles

As shown in Table 3, a statistically significant (*p* < 0.0001) decrease in the TC serum levels was found in Group V (treated with Repaglinide-loaded nanoparticles) (2.06 ± 0.14 mmol/L) when compared with the positive control group (Group II) (4.06 ± 0.24 mmol/L). On comparing the ability of ATC marketed tablets and Repaglinide marketed tablets to decrease the serum levels of TC with the positive control group, the results of THE ATC tablets (Group III) were not significant with a *p*-value of 0.4589. Meanwhile, the results obtained by the Repaglinide marketed tablets (Group IV) were significant with a *p*-value of 0.0038 [31].

Similar results were observed by a statistically significant decrease in the serum levels of TG and LDLs when comparing the results for the rats in Group V (treated with Repaglinide-loaded nanoparticles) to the positive control Group II with a *p*-value <0.0001. On comparing the ability of the ATC marketed tablets and the Repaglinide marketed tablets to decrease the serum levels of TG with the positive control group, a statistically significant decrease in TG was observed at a *p*-value of <0.0001.

On the other hand, a statistically significant increase in the serum levels of HDLs was found when comparing the results between the rats in Group V and the positive control Group II with a *p*-value of 0.0074. The ATC marketed tablets and Repaglinide marketed tablets showed an increase in the serum levels of HDLs with the positive control group; the results were also non-significant, with *p*-values of 0.8781 and 0.2442, respectively.

The average weight of the rats at the beginning of the experiment was 200 ± 50 g. After the induction of hyperlipidemia for one week, there was an increase in the rats’ average body weight, which was calculated to be 300 ± 50 g. The rats in Group V were reweighed at the end of the experiment after the completion of the treatment, and their average weight was 280 ± 50 g, which indicates a decrease in their body weight after treatment with Repaglinide nanoparticles. Repaglinide and Atorvastatin have proved their ability to decrease the body weight of rats after treatment [63,64].

Finally, it was concluded that Group V, treated with Repaglinide nanoparticles (F5), showed the highest decrease in the serum levels of TC, TG, and LDLs among the treated groups, and this group also showed the highest increase in the level of HDLs. All of the results of the Repaglinide-treated groups were found to be significant.

## 4. Conclusions

Repaglinide-loaded albumin nanoparticles were successfully prepared using a desolvation technique. The formula was optimized using a 2^3^ full factorial design by studying the effect of the dependent variables, albumin% (A), acetone volume (B), and 1 µL glutaraldehyde/2 mg albumin (C). The effects of these variables were assessed at two different levels on the dependent responses, namely particle size (Y1), zeta potential (Y2), and entrapment efficiency (Y3). The optimized formula (F5) was selected based on the criteria of maximizing the EE% and minimizing the PS. The optimized formula (F5) showed acceptable values of EE%, PS, PDI, and ZP, and the homogenous and spherical shape of the vesicles was confirmed by TEM imaging. A smooth external surface with no drug crystals on the surface was found based on the SEM study. Molecular docking simulation showed a strong affinity to several protein targets, and the results were very promising, where Repaglinide gave a score of −7.70 Kcal/mol compared to Mevastatin (−6.71 Kcal/mol) and Atorvastatin (−8.36 Kcal/mol). This in vivo study demonstrated a statistically significant decrease in the serum levels of TC, LDLs, and TG in Group V of rats treated with Repaglinide-loaded nanoparticles when compared with the positive control group (Group II), with a *p*-value of < 0.000, and a statistically significant increase (*p*-value of < 0.0001) in the serum levels of HDLs was recorded. In conclusion, BSA nanoparticles could be novel carriers for the parenteral delivery of Repaglinide to sustain drug release and enhance its antihyperlipidemic effect, in addition to its antihyperglycemic effect, especially in elderly patients, to eliminate hazards following the use of statins.

## Figures and Tables

**Table 1:** Independent variables and dependent responses.

In-Dependent Variables	High Level (+1)	Low Level (−1)	Dependent Responses	Goal
(A) Albumin *v*/*v* %	1	0.5	Particle size nm (Y1)	Minimize
(B) Acetone volume (mL)	20	10	Zeta potential mV (Y2)	Maximize
(C) µL Glutraldehyde/2 mg albumin	3	1	Entrapment efficiency % (Y3)	Maximize

A, albumin concentration (%*v*/*v*); B, acetone volume (mL); C, µL Glutraldehyde/2 mg albumin; Y1, particle size (PS); Y2, zeta potential (ZP); Y3, entrapment efficiency (EE %).

**Table 2:** Composition of prepared albumin–Repaglinide nanoparticles (F1–F8) and the observed responses.

Formula	A	B	C	Particle Size (nm) (Y1)	Zeta Potential (mV) (Y2)	Entrapment Efficiency (%) (Y3)
F1	0.5	10	1	143.30 ± 0.63	−27.53 ± 0.46	65.12 ± 0.21
F2	0.5	20	1	183.06 ± 0.91	−26.53 ± 0.29	69.77 ±0.24
F3	0.5	10	3	136.63 ±0.55	−29.63 ± 0.57	61.03 ± 0.64
F4	0.5	20	3	166.36 ± 0.72	−29.63 ± 0.25	68.91 ± 0.60
F5	1	10	1	181.86 ± 0.71	−24.26 ± 0.21	76.37 ± 0.75
F6	1	20	1	217.83 ± 0.43	−22.43 ± 0.31	86.90 ± 1.04
F7	1	10	3	178.33 ± 0.73	−25.43 ± 0.56	70.50 ± 0.36
F8	1	20	3	201.96 ±0.69	−28.73 ± 1.21	82.43 ± 1.24

(A), albumin%; (B), mL of acetone; (C) Glutaraldehyde µL/2 mg albumin.

**Table 3:** Serum blood levels of TC, TG, HDLs, and LDLs in different rat groups.

Groups	TC (mmol/L)	TG (mmol/L)	LDLs (mmol/L)	HDLs (mmol/L)
Group I	1.41 ± 0.07	0.94 ± 0.04	0.25 ± 0.02	1.00 ± 0.31
Group II	4.06 ± 0.24	2.24 ± 0.10	0.94 ± 0.04	0.52 ± 0.07
Group III	3.20 ± 0.49	1.10 ± 0.32	0.66 ± 0.03	0.60 ± 0.03
Group IV	3.70 ± 0.60	0.95 ± 0.28	0.51 ± 0.03	0.70 ± 0.07
Group V	2.06 ± 0.14	0.80 ± 0.02	0.39 ± 0.01	0.84 ± 0.04

Group I is a normal group (negative control), Group II represents hyperlipidemic non-treated rats (positive control), Group III represents rats treated with Repaglinide oral marketed tablets, Group IV represents rats treated with ATC marketed tablets, and Group V represents rats treated with the selected RG-loaded nanoparticles (F5).

## Data Availability

The original contributions presented in this study are included in the article; further inquiries can be directed to the corresponding authors.

## Supplementary Materials

The following supporting information can be downloaded at: https://www.mdpi.com/article/10.3390/pharmaceutics17030350/s1, Table S1: ANOVA statistical analysis of responses (Y1, Y2, and Y3); Table S2: Kinetic release study of different formulations of Repaglinide-loaded BSA nanoparticles (F1-F8); Figure S1: a. (A) Mevastatin; (B) Atorvastatin; (C) Repaglinide; (D) interaction types: 2D and 3D interaction diagrams with HMG-CoA reductase pocket (PDB: 1HW8). b. (A) Nevanimibe; (B) Repaglinide; (C) interaction types: 2D and 3D interaction diagrams with acetyl Co-A acyltransferase (PDB: 7N6R). c. 2D and 3D interaction diagrams of nominated compounds (A) Ezetimibe; (B) Repaglinide in the active site of NPC1L1 (PDB: 6V3H).

## Abbreviations

The following abbreviations are used in this manuscript:

ACAT	Acyl-CoA cholesterol acyl transferase
ATC	Atorvastatin calcium
BSA	Bovine serum albumin
CDC	Center for Disease Control and Prevention
CETP	Cholesterol ester transfer protein
DSC	Differential scanning calorimetry
EE	Entrapment efficiency
HSA	Human serum albumin
HDLs	High-density lipoproteins
HMG-CoA	β-Hydroxy β-methylglutaryl-CoA
IACUC	Institutional Animal Care and Use Committee
LDLs	Low-density lipoproteins
NPC1L1	Niemann-Pick C1 Like 1
PS	Particle size
PDB	Protein data bank
PDI	Polydispersibility index
PPARs	Peroxisome proliferation-activated receptors
SEMs	Scanning electron microscopes
SD	Standard deviation
SPB	Sörenson’s phosphate buffer
TC	Total cholesterol
TGs	Triglycerides
TEMs	Transmission electron microscopes
ZP	Zeta potential
