# Supplementary material for: Albumin-Based Nanoparticles with Factorial Design as a Promising Approach for Remodeled Repaglinide: Evidence from In Silico, In Vitro, and In Vivo Evaluations
Source: Pharmaceutics. 2025 Mar 9;17(3):350. doi: 10.3390/pharmaceutics17030350 (PMC11946440; doi:10.3390/pharmaceutics17030350)
Supplement: Supplementary file 1 [file pharmaceutics-17-00350-s001.zip › pharmaceutics-3495995-supplementary.pdf]

Table S1. ANOVA statistical analysis of responses (Y1, Y2, and Y3).

**Source**	**Y1 response**			**Y2 response**			**Y3 response**		
	**F value**	**P value**	**Significance**	**F value**	**P value**	**Significance**	**F value**	**P value**	**Significance**
**Model**	4885.66	<0.0001	Significant	12.44	<0.0001	Significant	479.11	<0.0001	Significant
**A**	15912.51	<0.0001	Significant	31.93	< 0.0001	Significant	1789.14	< 0.0001	Significant
**B**	11688.23	<0.0001	Significant	0.051	0.8239	Non Significant	831.08	< 0.0001	Significant
**C**	1282.65	<0.0001	Significant	32.61	< 0.0001	Significant	155.17	< 0.0001	Significant
**AB**	68.73	<0.0001	Significant	1.28	0.2741	Non Significant	65.23	< 0.0001	Significant
**AC**	11.03	0.0040	Significant	1.02	0.3269	Non Significant	20.41	0.0003	Significant
**BC**	350.83	<0.0001	Significant	7.77	0.0126	Significant	13.66	0.0018	Significant

Table S2. Kinetic release study of different formulations Repaglinide-loaded BSA nanoparticles (F1-F8).

Formulations	**Zero order**	**First order**	**Higuchi**	**Hixson-Crowell**	**Korsmeyer-Peppas**	
	**R^2**	**R^2**	**R^2**	**R^2**	**R^2**	**n**
F1	0.5134	0.9899	-0.3038	-0.5331	0.8648	0.126
F2	0.6683	0.9009	-0.5413	-0.7401	0.9321	0.114
F3	0.6788	0.7610	-0.8866	-0.7345	0.9417	0.1
F4	0.7737	0.3163	-0.3064	-0.7004	0.9506	0.125
F5	0.6365	0.9210	-0.7575	0.7349	0.9356	0.093
F6	0.6497	-0.5330	0.7689	0.7537	0.9516	0.094
F7	0.6450	0.7665	0.7510	0.7316	0.9689	0.082
F8	0.5256	-0.2620	0.6413	0.6045	0.9827	0.051

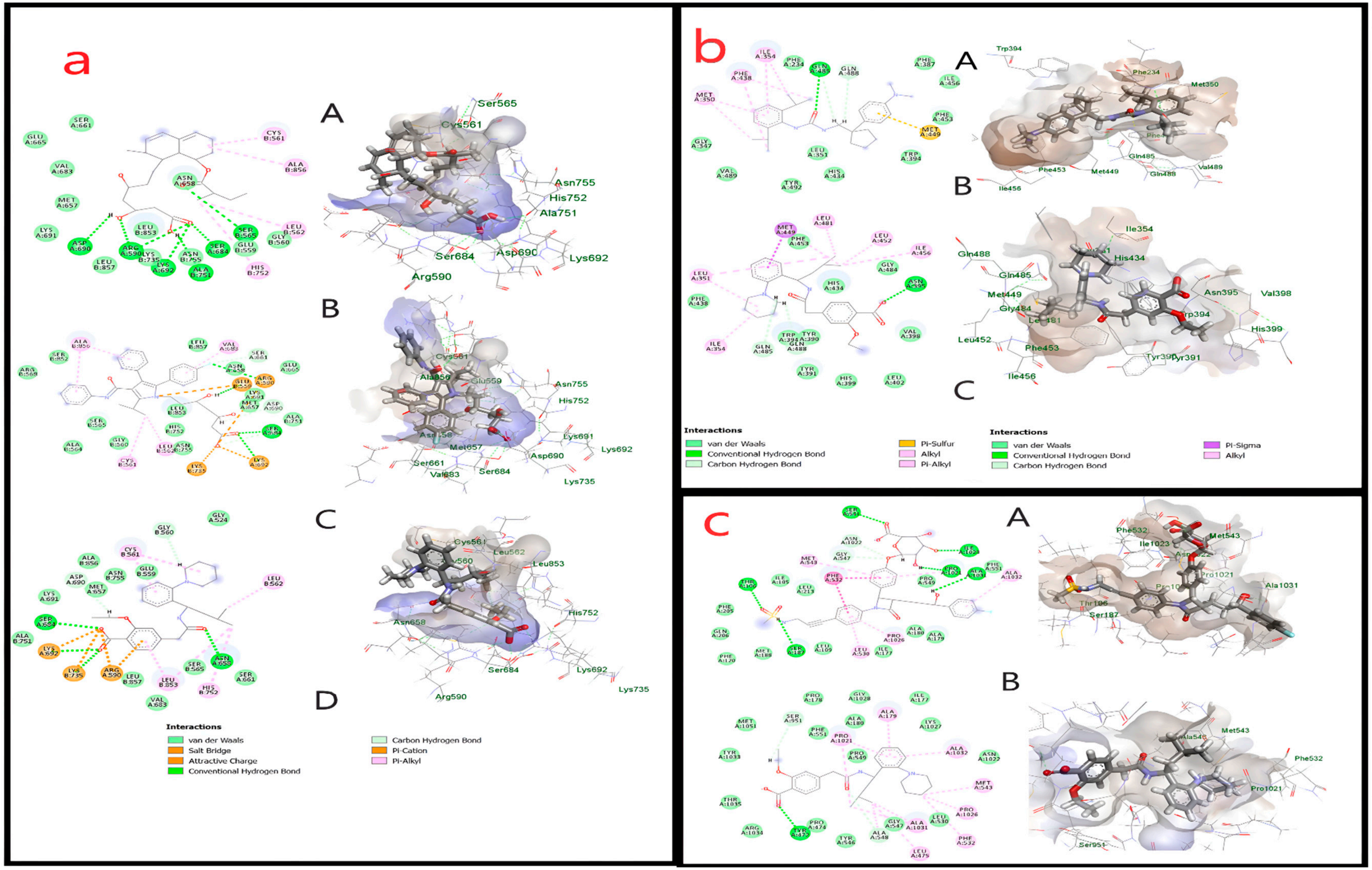

Figure S1. a. A) Mevastatin; B) Atorvastatin; C) Repaglinide; D) interaction types; 2D and 3D interaction diagrams with HMG-CoA reductase pocket (PDB: 1HW8). **b.** A) Nevanimibe; B) Repaglinide; C) interaction types; 2D and 3D interaction diagrams with Acetyl Co-A acyltransferase (PDB: 7N6R). **c.** 2D and 3D interaction diagrams of nominated compounds A) Ezetimibe; B) Repaglinide in the active site of NPC1L1 (PDB: 6V3H).
